# *PTGER4* promoter methylation may serve as an independent prognostic biomarker and is associated with response to hypomethylating agents in pediatric acute myeloid leukemia

**DOI:** 10.3389/fphar.2026.1922446

**Published:** 2026-09-08

**Authors:** Yu-juan Xue, Xiaoyu Cao, Yinping Yang, Yu Wang, Fangyuan Zheng, Ai-dong Lu, Yue-ping Jia, Le-ping Zhang, Hui-min Zeng

**Affiliations:** 1 Department of Pediatrics, Peking University People’s Hospital, Peking University, Beijing, China; 2 Academic Department, Beijing Jahon Vast Tech Co., Ltd., Beijing, China

**Keywords:** epigenetics, hypomethylating agents, pediatric acute myeloid leukemia, personalized medicine, PTGER4 methylation, tumor biomarkers

## Abstract

**Background:**

Despite advances in therapy, 30%–40% of pediatric acute myeloid leukemia (AML) patients still experience treatment failure due to relapse or toxicity. Current risk stratification based on genetic features remains insufficient, and epigenetic biomarkers for pediatric AML are understudied. This study aimed to evaluate the prognostic and predictive value of *PTGER4, SHOX2*, and *HOXA9* promoter methylation in pediatric AML.

**Methods:**

We enrolled 69 newly diagnosed pediatric AML patients treated with standardized chemotherapy. Promoter methylation levels were measured by multiplex methylation-specific PCR. Associations between methylation status and clinical characteristics, survival outcomes, and response to hypomethylating agents (HMAs) were analyzed using univariate and multivariate Cox regression, sensitivity analyses, and subgroup validation.

**Results:**

Methylation levels of all three genes were significantly associated with age and core binding factor abnormalities. *PTGER4* methylation positivity was identified as an independent adverse prognostic factor for event-free survival (hazard ratio (HR) = 3.456, 95% CI 1.126–10.610, *P* = 0.030), with a significant dose-response relationship between methylation levels and survival. Subgroup analyses confirmed its prognostic value in patients with platelet count <50 × 10^9^/L, those achieving complete remission (CR) after induction and patients within transplant group. Among *PTGER4* methylation-positive patients, HMA-based therapy was associated with significantly improved 3-year overall survival (84.6% vs. 19.0%, *P* = 0.011).

**Conclusion:**

*PTGER4* promoter methylation is a promising prognostic biomarker for pediatric AML and correlates with improved response to HMAs. It could help refine risk stratification and guide individualized treatment decisions, particularly in patients with baseline thrombocytopenia and those achieving post-induction CR or receiving transplant.

## 1 Introduction

Acute myeloid leukemia (AML) is a heterogeneous hematological malignancy that accounts for approximately 20% of childhood leukemia cases and 30% of pediatric leukemia-related deaths worldwide (Umeda et al., 2025). Despite advances in intensive chemotherapy and hematopoietic stem cell transplantation (HSCT) over the past 2 decades, 30%–40% of pediatric AML patients still experience treatment failure due to relapse or treatment-related toxicity (Dohner et al., 2022).

Current risk stratification systems rely primarily on recurrent fusion genes and somatic mutations. However, these genetic markers fail to accurately predict outcomes in a significant proportion of patients, highlighting an urgent unmet need for novel reliable biomarkers (Umeda et al., 2024). Epigenetic alterations, particularly DNA methylation, play a critical role in leukemogenesis and have emerged as promising biomarkers in many adult malignancies (Nishiyama and Nakanishi, 2021). Promoter hypermethylation of tumor suppressor genes is a well-recognized hallmark of cancer initiation and progression. In solid tumors, DNA methylation detection has been widely adopted as a promising approach for auxiliary diagnosis, prognostic assessment and follow-up monitoring (Dolcini et al., 2025; Lee et al., 2024; Yu et al., 2023). While aberrant DNA methylation has been extensively studied in adult hematological malignancies (Li et al., 2025; Aryal and Lu, 2024), its role in pediatric AML remains poorly characterized. To date, few studies have systematically evaluated the prognostic value of specific methylation markers in pediatric AML, and none have investigated their potential as predictive biomarkers for hypomethylating agent (HMA) response in this population.

In this study, we measured the promoter methylation levels of three candidate genes (*SHOX2, HOXA9*, and *PTGER4*) in bone marrow samples from 69 newly diagnosed pediatric AML patients. The primary objectives were: (1) to evaluate the prognostic value of these methylation markers for overall survival (OS) and event-free survival (EFS); (2) to identify clinical subgroups where these markers have the greatest utility; and (3) to explore whether *PTGER4* methylation status predicts response to HMA-based therapy. Our findings provide evidence that *PTGER4* promoter methylation may serve as a clinically useful biomarker for risk stratification and personalized treatment in pediatric AML.

## 2 Materials and methods

### 2.1 Study design and subjects

This retrospective cohort study was conducted at the Department of Pediatric Hematology of Peking University People’s Hospital. The study was approved by the Ethics Committee of Peking University People’s Hospital (2025PHB324-001), and written informed consent was obtained from all guardians in accordance with the Declaration of Helsinki.

A total of 69 consecutive newly diagnosed pediatric AML patients treated between September 2020 and December 2023 were enrolled. Inclusion criteria: (1) age <18 years at diagnosis; (2) confirmed diagnosis of *de novo* AML according to WHO 2016 criteria; (3) received standardized induction and consolidation chemotherapy at our institution. Exclusion criteria: (1) acute promyelocytic leukemia; (2) relapsed AML; (3) prior treatment at other institutions; (4) insufficient bone marrow DNA for methylation analysis.

Bone marrow samples were collected at diagnosis before any treatment, and excess DNA was stored at −80 °C. Twenty-one bone marrow samples from healthy hematopoietic stem cell donors were used as controls. All patients were followed up until 31 December 2025. No missing clinical or follow-up data were present in the cohort.

### 2.2 Clinical and molecular detection

Routine blood and biochemical tests were performed using Sysmex XN-5000 and Roche cobas 8000 analyzers. Bone marrow samples were analyzed for morphology, multiparameter flow cytometry (MFC) immunophenotyping (BD FACSCanto II), and G-banding karyotyping. Recurrent fusion genes and somatic mutations were detected by reverse transcription polymerase chain reaction (RT-PCR), Sanger sequencing, and fluorescence *in situ* hybridization (FISH) according to standard laboratory protocols.

### 2.3 Gene methylation detection

Genomic DNA was extracted from bone marrow mononuclear cells using the QIAamp DNA Blood Mini Kit (Qiagen, Hilden, Germany). Sodium bisulfite modification was performed using the EZ DNA Methylation-Gold Kit (Zymo Research, Irvine, CA, United States) according to the manufacturer’s instructions, as described in previous studies (Xie et al., 2018; Lu et al., 2022).

Promoter methylation levels of *SHOX2, HOXA9*, and *PTGER4* were measured by multiplex methylation-specific real-time PCR (MS-PCR) using a commercial kit (Beijing Jahon Vast Tech Co., Ltd., Beijing, China). *β-ACTB* was used as an internal reference to normalize DNA input. Primer sequences targeting the promoter CpG islands were provided by the kit manufacturer and have been previously validated for clinical use.

The amplification conditions were: 95 °C for 10 min; 45 cycles of 95 °C for 30 s, 58 °C for 35 s, 72 °C for 30 s; and a final extension at 72 °C for 8 min. Methylated plasmids and nuclease-free water were included as positive and negative controls in each run. Cycle threshold (CT) values were used to quantify methylation levels, with lower CT values indicating higher methylation.

The optimal cutoff values for methylation positivity were determined by receiver operating characteristic (ROC) curve analysis using the maximum Youden index. Leave-one-out cross-validation was performed to confirm the stability of the cutoffs.

### 2.4 Treatment regimens

All patients received standardized pediatric AML chemotherapy regimens.

- Induction therapy: DAE (daunorubicin + cytarabine + etoposide);
- Consolidation therapy: 2–4 cycles of risk-stratified chemotherapy after complete remission (CR);
- Allogeneic HSCT: Recommended for high-risk patients (including adverse cytogenetics, induction failure, or persistent minimal residual disease (MRD));
- Individualized intensified therapy: HMAs were administered to patients with high-risk clinical features as determined by pediatric hematology-oncology committee of this center, including adverse cytogenetics, induction failure, or persistent minimal residual disease (MRD) ≥10^−2^ after two cycles of chemotherapy. All HMA-containing regimens were delivered during post-induction consolidation therapy, with administration cycles determined by clinical risk stratification and tolerance. HMAs used in this cohort included azacitidine and decitabine. Azacitidine was administered subcutaneously at 75 mg/m^2^/day for seven consecutive days per consolidation cycle; decitabine was given via 1-h intravenous infusion at 20 mg/m^2^/day for five consecutive days per cycle. In total, 33 patients (47.8%) received HMAs during consolidation therapy. Among them, eight patients received HMAs alone (azacitidine, n = 3; decitabine, n = 5), and 25 patients further received combination therapy with oral *BCL-2* inhibitor venetoclax (Ven, 100–400 mg/m^2^; maximum dose: 400 mg/day; if well tolerated, administer continuously for 14 days per cycle). No patient received venetoclax as monotherapy. Three patients with high allelic ratio *FLT3-ITD* mutation received FLT3 tyrosine kinase inhibitors in combination with chemotherapy.

### 2.5 Definition and assessments

Bone marrow assessment was performed on day 21–23 after initiation of the induction course, coinciding with hematologic recovery. MRD was detected by MFC with a sensitivity of 10^−4^ (analysis of 1,000,000 viable cells required for MRD negativity) (Zhao et al., 2017). CR was defined as <5% leukemic blasts in bone marrow with normal peripheral blood counts, and no extramedullary disease. Relapse was defined as hematological or extramedullary recurrence of leukemia.

The primary endpoints were OS and EFS. OS was calculated from diagnosis to death from any cause or last follow-up. EFS was calculated from diagnosis to the first event (relapse, second malignancy, or death) or last follow-up.

### 2.6 Statistical analysis

Statistical analyses were performed using SPSS 26.0 (SPSS Inc., Chicago, IL, United States) and GraphPad Prism 10.0. Categorical variables were compared using the χ^2^ test or Fisher’s exact test. Continuous variables were compared using the Mann-Whitney U test. Spearman’s rank correlation test was used to assess correlations between continuous variables and methylation CT values.

Receiver operating characteristic (ROC) curve analyses were performed to derive cohort-specific optimal thresholds of baseline platelet count and post-induction MRD for predicting OS and EFS events. Binary survival status was defined as the outcome variable, and continuous laboratory values were used as predictive variables. The threshold yielding the maximum Youden index was regarded as the data-driven optimal cutoff.

For post-induction MRD, ROC analysis identified an optimal value of 0.79%, which was highly consistent with the clinically adopted stratification cutoff of 1% (10^−2^). Given the lack of universal standardized MRD cutoffs across time points in pediatric AML studies, we retained the widely cited 1% boundary to ensure comparability with published cohorts.

For baseline platelet count, the ROC-derived optimal cutoff was 52 × 10^9^/L, closely matching the validated literature threshold of 50 × 10^9^/L for high-risk thrombocytopenia in pediatric AML. We retained the published 50 × 10^9^/L cutoff rather than the cohort-specific value to maximize the generalizability of our subgroup findings.

Survival curves were constructed using the Kaplan-Meier method, and differences were compared using the Log-rank test. Variables with a P-value <0.1 in univariate analysis were included in multivariate Cox proportional hazards regression models to identify independent prognostic factors.

Sensitivity analyses were performed to confirm the robustness of the results: (1) patients were stratified into three groups based on *PTGER4* methylation percentiles to assess dose-response relationships; (2) multivariate Cox regression was repeated using continuous *PTGER4* CT values instead of binary methylation status.

To address potential confounding by indication in HMA treatment assignment while avoiding overfitting given the limited sample size of the PTGER4-positive subgroup, we constructed two clinically meaningful composite variables and included them in multivariate Cox proportional hazards regression: (1) adverse early treatment response (defined as non-CR after induction therapy or MRD ≥10^−2^ after two cycles of chemotherapy); (2) adverse cytogenetic abnormalities. This model was used to evaluate the independent effect of HMA therapy on overall survival in *PTGER4* methylation-positive patients. All statistical tests were two-sided, and a *P* value <0.05 was considered statistically significant.

## 3 Results

### 3.1 Baseline clinical characteristics

**FIGURE 1:**
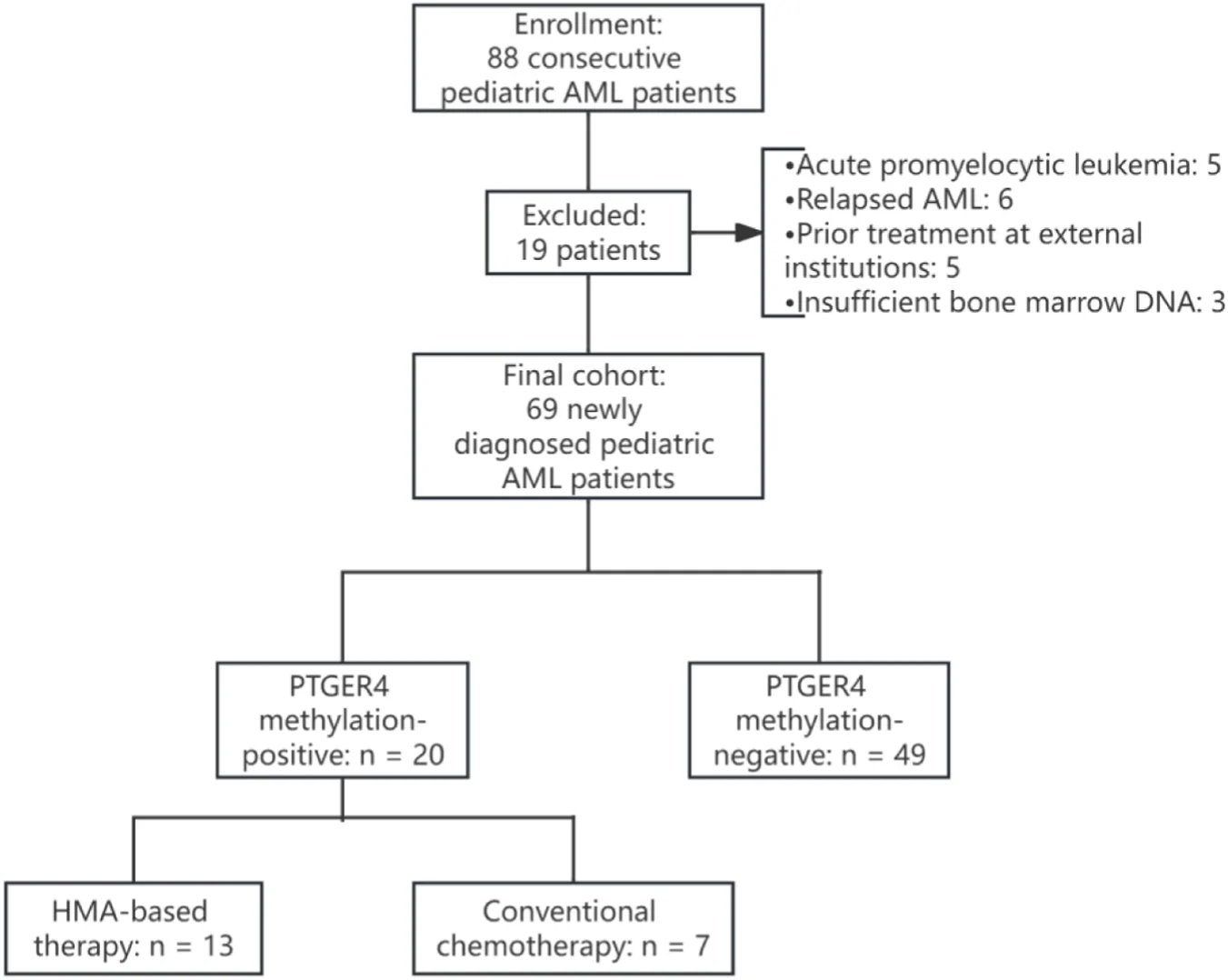
Patient enrollment flowchart. AML, acute myeloid leukemia; HMA, hypomethylating agent.

The patient enrollment process is summarized in Figure 1. A total of 88 consecutive pediatric AML patients were initially screened between September 2020 and December 2023. After exclusions (5 with acute promyelocytic leukemia, 6 with relapsed AML, five treated at other institutions, and 3 with insufficient bone marrow DNA for methylation analysis), 69 newly diagnosed *de novo* pediatric AML patients were finally included in the study cohort. Among them, 20 patients were *PTGER4* methylation-positive, of whom 13 received HMA-based therapy and seven received conventional chemotherapy. The baseline characteristics of the 69 enrolled patients are summarized in Table 1. The median age was 9.0 years (range, 0.6–16.0 years), and 62.3% were male. The median initial white blood cell count (WBC) was 10.3 × 10^9^/L, the median hemoglobin was 82.0 g/L, and the median platelet count was 50.0 × 10^9^/L. Cytogenetic abnormalities included *CBF* rearrangements (31.9%), *KMT2A* rearrangements (21.7%), and other rare aberrations.

**TABLE 1:** Clinical and genetic characteristics of pediatric AML patients stratified by *SHOX2, HOXA9,* and *PTGER4* methylation status.

Characteristic	AML N(%)	SHOX2+ (%)	SHOX2- (%)	X^2^	P	HOXA9+ (%)	HOXA9- (%)	X^2^	P	PTGER4+ (%)	PTGER4- (%)	X^2^	P
Total	69 (100.0)	16 (23.2)	53 (76.8)	–	–	20 (29.0)	49 (71.0)	–	–	20 (29.0)	49 (71.0)	–	–
Age (years)				4.350	0.037			12.162	<0.001			3.329	0.110
Median (range)	9.0 (0.6–16)	11.0 (4.0–15.0)	8.0 (0.6–16.0)			13.0 (1.0–15.0)	6.0 (0.6–16.0)			4.0 (0.6–15)	10.0 (1.0–10.0)		
<9	33 (47.8)	4 (25.0)	29 (54.7)			3 (15.0)	30 (61.2)			13 (65.0)	20 (40.8)		
≥9	36 (52.2)	12 (75.0)	24 (45.3)			17 (85.0)	19 (38.8)			7 (35.0)	29 (59.2)		
Sex				0.000	0.986			0.642	0.585			1.929	0.185
Female	26 (37.7)	6 (37.5)	20 (37.7)			9 (45.0)	17 (34.7)			5 (25.0)	21 (42.9)		
Male	43 (62.3)	10 (62.5)	33 (62.3)			11 (55.0)	32 (65.3)			15 (75.0)	28 (57.1)		
WBC (×10^9^/L)				-	1.000			–	1.000			–	1.000
Median (range)	10.3 (0.88–228.5)	5.0 (0.88–186.9)	13.0 (1.7–228.5)			4.2 (0.88–201.65)	13 (1.7–228.5)			13.5 (1.5–228.5)	9.1 (0.88–203.0)		
<50	59 (85.6)	14 (87.5)	45 (84.9)			17 (85.0)	42 (85.7)			17 (85.0)	42 (85.7)		
≥50	10 (14.4)	2 (12.5)	8 (15.1)			3 (15.0)	7 (14.3)			3 (15.0)	7 (14.3)		
Hb (g/L)				–	0.032			–	0.902			–	0.364
Median (range)	82.0 (34.0–140.0)	71.0 (35.0–111)	84.0 (34.0–140.0)			72.5 (46.0–140.0)	82.0 (34.0–126.0)			83.0 (42.0–126.0)	80.0 (34.0–140.0)		
PLT (×10^9^/L)				–	0.541			–	0.166			–	0.997
Median (range)	50.0 (6.0–360.0)	60.0 (7.0–302.0)	47.0 (6.0–360.0)			47.0 (10.0–360.0)	50.0 (6.0–211.0)			66.0 (7.0–202.0)	44.0 (6.0–360.0)		
Cytogenetic abnormality													
With *CBF*	22 (31.9)	1 (6.3)	21 (39.6)	6.302	0.014	8 (40.0)	14 (28.6)	0.854	0.401	1 (5.0)	21 (42.9)	9.372	0.003
With11q23/*KMT2A-r*	15 (21.7)	3 (18.8)	12 (22.6)	0.109	0.741	1 (5.0)	14 (28.6)	–	0.051	3 (15.0)	12 (24.5)	0.297	0.585
*EVI1*	6 (8.7)	2 (12.5)	4 (7.5)	–	0.617	2 (10.0)	4 (8.2)	–	1.000	3 (15.0)	3 (6.1)	–	0.346
*NUP98-NSD1*	3 (4.3)	3 (18.8)	0 (0)	–	0.011	0 (0.0)	3 (6.1)	–	0.551	2 (10.0)	1 (2.0)	–	0.199
*FLT3-ITD*	3 (4.3)	1 (6.3)	2 (3.8)	–	0.553	2 (10.0)	1 (2.0)	–	0.199	2 (10.0)	1 (2.0)	–	0.199
*FLT3-ITD*	3 (4.3)	1 (6.3)	2 (3.8)	–	0.553	2 (10.0)	1 (2.0)	–	0.199	2 (10.0)	1 (2.0)	–	0.199
*NPM1*	2 (2.9)	1 (6.3)	1 (1.9)	–	0.413	1 (5.0)	1 (2.0)	–	0.499	0 (0.0)	2 (4.1)	–	1.000
*CEBPA*	2 (2.9)	0 (0)	2 (3.8)	–	1.000	2 (10.0)	0 (0.0)	–	0.081	0 (0.0)	2 (4.1)	–	1.000
Extramedullary involvement	6 (8.7)	1 (6.3)	5 (9.4)	–	1.000	1 (5.0)	5 (10.2)	–	0.664	2 (10.0)	4 (8.2)	–	1.000
CR after IT	57 (82.6)	14 (87.5)	43 (81.1)	–	0.718	16 (80.0)	41 (83.7)	–	0.734	15 (75.0)	42 (85.7)	–	0.309
MRD ≥10^-2^ after IT	21 (30.4)	5 (31.3)	16 (30.2)	–	1.000	8 (40.0)	13 (26.5)	1.217	0.387	8 (40.0)	13 (26.5)	1.217	0.387
HMAs±Ven	33 (47.8)	11 (68.8)	22 (41.5)	3.655	0.056	11 (55.0)	22 (44.9)	0.581	0.446	13 (65.0)	20 (40.8)	3.329	0.068
Follow-up time (m) Median (range)	43.5 (2.8–64.8)			–	–			–	–			–	–
HSCT at CR1	42 (60.9)	13 (81.3)	29 (54.7)	3.632	0.080	7 (35.0)	35 (71.4)	7.913	0.007	17 (85.0)	25 (51.0)	6.885	0.013

AML, acute myeloid leukemia; WBC, white blood cell; Hb, hemoglobin; PLT, platelet; *CBF*, core binding factor; *KMT2A-r*, lysine methyltransferase 2A gene rearrangement; CR, complete remission; IT, induction therapy; MRD, minimal residual disease; HMAs, hypomethylating agents; Ven, venetoclax; m, month; HSCT, hematopoietic stem cell transplantation; CR1, first complete remission. (+), methylation-positive; (−), methylation-negative.

Fifty-seven patients (82.6%) achieved CR after induction therapy, and 21 (30.4%) had post-induction MRD ≥10^−2^. Thirty-three patients (47.8%) received HMA-containing regimens, and 42 (60.9%) underwent allo-HSCT at first CR. The median follow-up time was 43.5 months (range, 2.8–64.8 months).

### 3.2 Methylation detection and cutoff determination

All bone marrow DNA samples passed quality control, with concentrations ranging from 20.9 to 1,216.0 ng/μL (Supplementary Figure S1). CT values for *SHOX2, HOXA9,* and *PTGER4* showed continuous heterogeneous distribution across patients.

**FIGURE 2:**
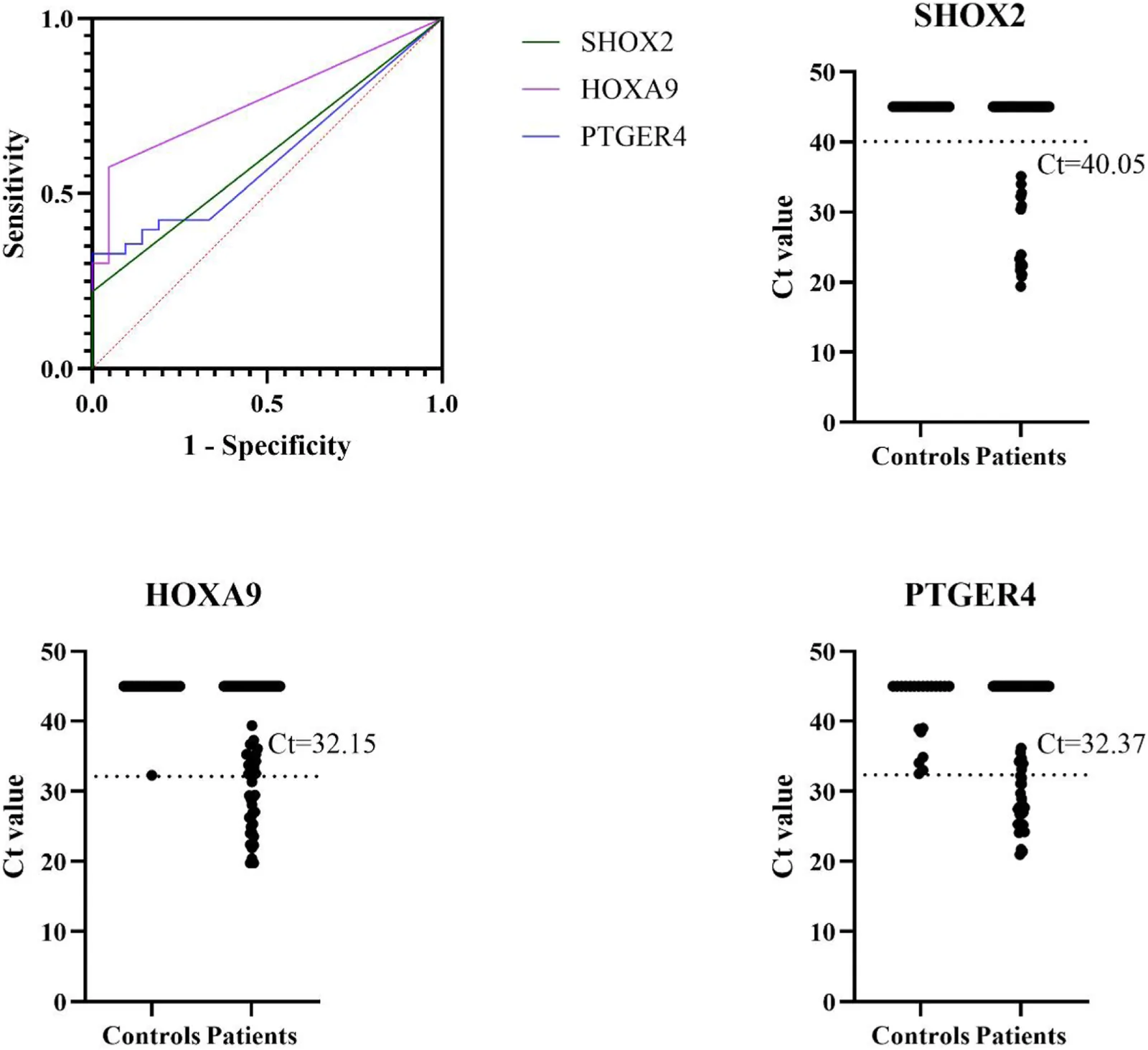
ROC curves and Ct value distribution for the establishment of optimal cut-off values of *SHOX2, HOXA9* and *PTGER4* methylation.

ROC curve analysis identified optimal cutoff values of 40.05 for *SHOX2*, 32.15 for *HOXA9*, and 32.37 for *PTGER4* (Figure 2). Leave-one-out cross-validation confirmed the stability of these cutoffs. Samples with CT values below the cutoff were defined as methylation-positive. The methylation-positive rates were 23.2% for *SHOX2*, 29.0% for *HOXA9*, and 29.0% for *PTGER4*.

### 3.3 Correlations between gene methylation and clinical characteristics

#### 3.3.1 Associations between binary methylation status and clinical features

**FIGURE 3:**
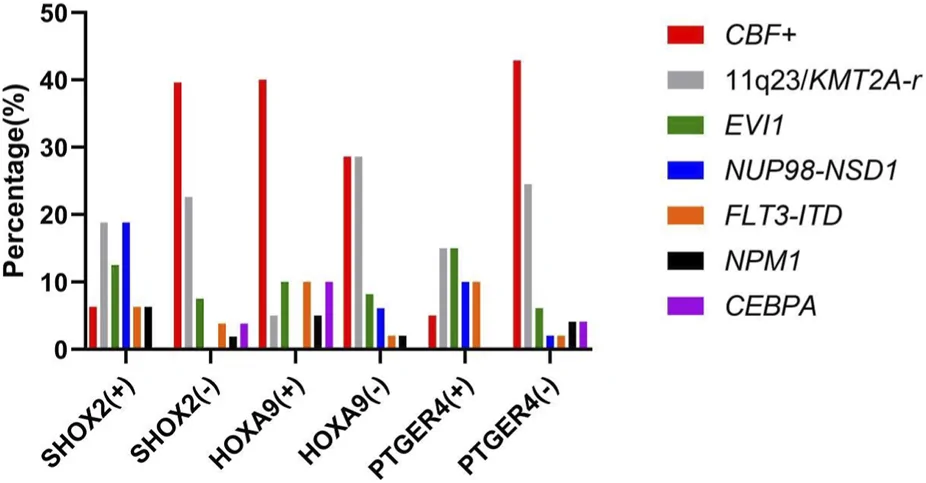
Associations of *SHOX2, HOXA9*, and *PTGER4* methylation statuses with molecular genetic abnormalities in pediatric AML patients. AML, acute myeloid leukemia; CBF, core binding factor; KMT2A-r, lysine methyltransferase 2A gene rearrangement; EVI1, ecotropic viral integration site 1; FLT3-ITD, FMS-like tyrosine kinase 3-internal tandem duplication; NPM1, nucleophosmin 1; CEBPA, CCAAT/enhancer binding protein alpha; (+), methylation-positive; (−), methylation-negative.

The methylation statuses of *SHOX2, HOXA9,* and *PTGER4* were dichotomized into methylation-positive and methylation-negative groups. Correlation analyses were performed to explore the associations of the binary methylation status of the three genes with baseline clinical, genetic characteristics and therapeutic response, treatment options in pediatric AML patients (Table 1; Figure 3; Supplementary Figure S2).

*SHOX2* methylation status was significantly associated with age, hemoglobin level, core binding factor (*CBF*) abnormalities, and *NUP98-NSD1* fusion gene status. *SHOX2* methylation-positive patients had a significantly higher median age than methylation-negative patients (11.0 years vs. 8.0 years, *P* = 0.010, Supplementary Figure S2). Specifically, 75.0% of patients in the *SHOX2* methylation-positive group were aged ≥9 years, compared with only 45.3% in the methylation-negative group (χ^2^ = 4.350, *P* = 0.037, Table 1). The median hemoglobin level was significantly lower in *SHOX2* methylation-positive patients than in methylation-negative patients (71.0 g/L vs. 84.0 g/L, *P* = 0.032, Supplementary Figure S2). For genetic characteristics, the prevalence of *CBF* abnormalities was significantly lower in the *SHOX2* methylation-positive group (6.3% vs. 39.6%, χ^2^ = 6.302, *P* = 0.014, Table 1). All patients with the *NUP98-NSD1* fusion gene were *SHOX2* methylation-positive, a statistically significant difference (*P* = 0.011, Table 1). No significant differences were observed between *SHOX2* methylation-positive and negative groups in terms of other features (all *P* > 0.05).

*HOXA9* methylation status was predominantly associated with age. *HOXA9* methylation-positive patients had a higher median age than methylation-negative cases (13.0 years vs. 6.0 years) (*P* < 0.001, Supplementary Figure S2). The proportion of patients aged ≥9 years was significantly higher in the *HOXA9* methylation-positive group (85.0%) than that in the negative group (38.8%) (χ^2^ = 12.162, *P* < 0.001, Table 1). Additionally, *HOXA9* methylation-positive patients had a significantly lower rate of allo-HSCT at first complete remission (CR1) (35.0% vs. 71.4%, χ^2^ = 7.913, *P* = 0.007, Table 1). No significant correlations were found between *HOXA9* methylation and other baseline clinical, laboratory, or genetic features (all *P* > 0.05).

*PTGER4* methylation status was significantly associated with age and genetic characteristics. The *PTGER4* methylation-positive group had a significantly higher median age than the negative group (*P* = 0.048, Supplementary Figure S2), whereas no significant difference was observed in the proportion of patients aged ≥9 years between the two groups. For genetic features, the prevalence of *CBF* abnormality was significantly lower in the *PTGER4* methylation-positive group than in the negative group (5.0% vs. 42.9%, χ^2^ = 9.372, *P* = 0.003, Table 1). In addition, *PTGER4* methylation-positive patients exhibited a significantly higher rate of allo-HSCT at CR1 (85.0% vs. 51.0%, χ^2^ = 6.885, *P* = 0.013, Table 1). Figure 3 visualizes the associations between the three gene methylation statuses and diverse molecular genetic abnormalities in pediatric AML.

#### 3.3.2 Correlation between continuous methylation CT values and clinical indicators

To preserve the full quantitative information of methylation levels and avoid potential bias from binary grouping, we further analyzed the associations between CT values of *SHOX2, HOXA9*, and *PTGER4* methylation and clinical characteristics (Supplementary Tables S1, S2).

As shown in Supplementary Table S1, significant differences in the overall distribution of CT values were observed between subgroups stratified by key molecular genetic abnormalities. For *SHOX2* methylation, patients with *CBF* abnormality had a significantly different CT value distribution compared with those without (median 45.0, IQR 45.0–45.0 vs. median 45.0, IQR 30.94–45.0; *P* = 0.009), indicating lower *SHOX2* methylation levels in the *CBF*-positive subgroup. For *HOXA9* methylation, patients with 11q23/*KMT2A* rearrangement exhibited significantly higher median CT values (45.00 vs. 35.27, *P* = 0.034), corresponding to lower *HOXA9* methylation levels. A borderline significant difference in *HOXA9* CT values was also observed between *CBF*-positive and negative subgroups (34.06 vs. 45.00, *P* = 0.052). For *PTGER4* methylation, the CT value distribution differed significantly between *CBF*-positive and negative patients (median 45.00, IQR 45.00–45.00 vs. median 45.00, IQR 27.74–45.00; *P* = 0.004), suggesting lower *PTGER4* methylation levels in the *CBF* subgroup. No significant intergroup differences in CT values of the three genes were detected between patients with and without CR after induction therapy, or between patients with and without MRD ≥10^−2^ after induction therapy (all *P* > 0.05).

Spearman correlation analysis further identified significant associations between continuous CT values of *SHOX2* and *HOXA9* and key clinical indicators (Supplementary Table S2). Age was significantly negatively correlated with *SHOX2* CT (r_s_ = −0.264, *P* = 0.028) and *HOXA9* CT (r_s_ = −0.479, *P* < 0.001), confirming that older age was associated with higher methylation levels of these two genes. WBC count was significantly positively correlated with *SHOX2* CT (r_s_ = 0.294, *P* = 0.014) and *HOXA9* CT (r_s_ = 0.301, *P* = 0.012), indicating that higher WBC counts corresponded to lower methylation levels of *SHOX2* and *HOXA9*. Hemoglobin level was significantly positively correlated with *SHOX2* CT (r_s_ = 0.252, *P* = 0.037), suggesting that lower hemoglobin levels were associated with higher *SHOX2* methylation levels. No significant correlations were found between platelet count and CT values of any of the three genes (all *P* > 0.05).

A significant positive correlation was observed between *SHOX2* and *HOXA9* methylation levels (r_s_ = 0.325, *P* = 0.006), indicating potential synergistic epigenetic regulation of these two genes in pediatric AML.

### 3.4 Survival analyses of three-gene methylation

#### 3.4.1 Kaplan-Meier survival analyses for binary methylation status

We further assessed the prognostic value of *SHOX2, HOXA9*, and *PTGER4* methylation status in pediatric AML.

**FIGURE 4:**
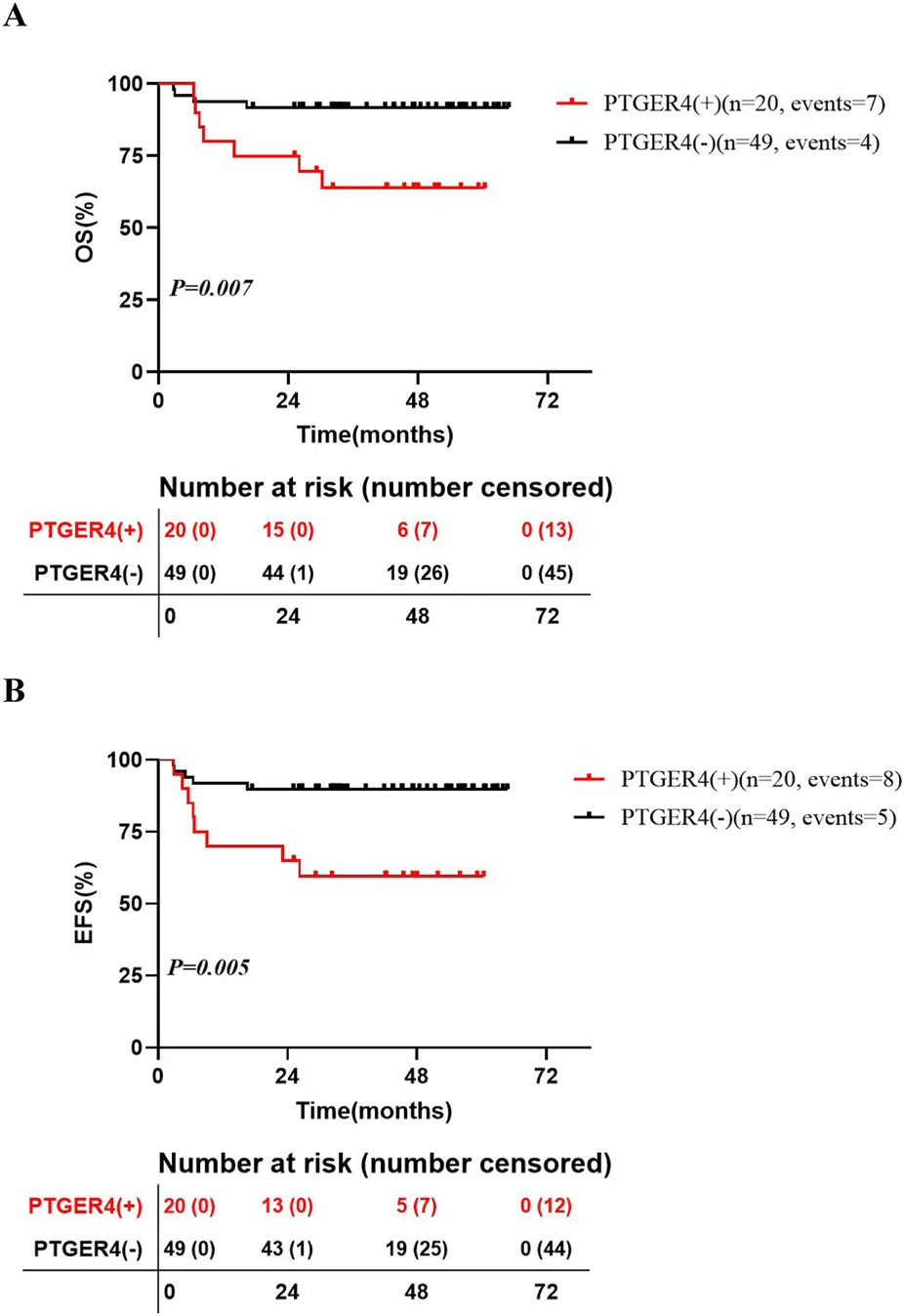
Kaplan-Meier curves of OS and EFS in pediatric AML patients stratified by *PTGER4* methylation status. **(A)** OS; **(B)** EFS. AML, acute myeloid leukemia; OS, overall survival; EFS, event-free survival; (+), methylation-positive; (−), methylation-negative.

Neither *SHOX2* nor *HOXA9* methylation was associated with OS or EFS (all *P* > 0.05). In contrast, *PTGER4* methylation-positive patients had significantly worse outcomes, with 3-year OS rates of 63.8% vs. 91.8% (χ^2^ = 7.256, *P* = 0.007) and 3-year EFS rates of 59.6% vs. 89.8% (χ^2^ = 8.038, *P* = 0.005) compared with methylation-negative patients. Survival curves are shown in Figure 4.

#### 3.4.2 Univariate and multivariate Cox regression analyses

Univariate and multivariate Cox proportional hazards regression models were constructed to identify independent prognostic factors for long-term survival (Table 2).

**TABLE 2:** Univariate and multivariate analysis of OS and EFS in AML group.

Variable	Comparison (Reference category)	OS Univariate (*P*)	OS Multivariate(*P*) HR(95%CI)	EFS Univariate (*P*)	EFS Multivariate(*P*) HR(95%CI)
Gender	Male vs Female (Ref: Female)	0.219	–	0.500	–
Age	Age ≥10y vs <10y (Ref: <10y)	0.162	–	0.185	–
WBC	WBC ≥50×10⁹/L vs <50×10⁹/L (Ref: <50)	0.680	–	0.886	–
Platelet	PLT ≥50×10⁹/L vs <50×10⁹/L (Ref: <50)	0.026	0.100 0.261(0.053–1.296)	0.006	0.016 0.156(0.034–0.708)
*SHOX2* methylation	*SHOX2*(+) vs *SHOX2*(−) (Ref: Negative)	0.713	–	0.984	–
*HOXA9* methylation	*HOXA9*(+) vs *HOXA9*(−) (Ref: Negative)	0.965	–	0.659	–
*PTGER4* methylation	*PTGER4*(+) vs *PTGER4*(−) (Ref: Negative)	0.007	0.117 2.742(0.776–9.687)	0.005	0.030 3.456(1.126–10.610)
*CBF*-AML	Present vs Absent (Ref: No *CBF*)	0.016	0.966 0(0–^∞^)	0.008	0.962 0(0–^∞^)
*KMT2A*-r	Present vs Absent (Ref: No *KMT2A-r*)	0.577	–	0.392	–
Post-induction remission	Non-CR vs CR (Ref: CR)	<0.001	0.004 5.665(1.722–18.641)	0.001	0.008 4.480(1.486–13.508)
Post-induction MRD	MRD ≥10^-2^ vs MRD <10^-2^ (Ref: MRD <10^-2^ )	0.009	0.468 2.299(0.243–21.768)	0.037	–
Extramedullary infiltration	Present vs Absent (Ref: Absent)	0.279	–	0.324	–
HMAs±Ven	Receive HMAs vs No HMAs (Ref: No)	0.548	–	0.535	–
HSCT	Undergo HSCT vs No HSCT (Ref: No)	0.807	–	0.930	–

AML, acute myeloid leukemia; OS, overall survival; EFS, event-free survival; WBC, white blood cell; CBF-AML, core binding factor-acute myeloid leukemia; CR, complete remission; MRD, minimal residual disease; HMAs, hypomethylating agents; Ven, venetoclax; HSCT, hematopoietic stem cell transplantation; HR, hazard ratio; CI, confidence interval; (+), methylation-positive; (−), methylation-negative.

Univariate analysis identified five significant OS prognostic factors: platelet count ≥50 × 10^9^/L (*P* = 0.026), *PTGER4* methylation positivity (*P* = 0.007), *CBF*-AML (*P* = 0.016), non-CR after induction (*P* < 0.001), and post-induction MRD ≥10^−2^ (*P* = 0.009). For EFS, significant factors included platelet count ≥50 × 10^9^/L (*P* = 0.006), *PTGER4* methylation positivity (*P* = 0.005), *CBF*-AML (*P* = 0.008), non-CR after induction (*P* < 0.001), and post-induction MRD ≥10^−2^ (*P* = 0.037). *SHOX2* and *HOXA9* methylation showed no prognostic significance (all *P* > 0.05).

Further multivariate model indicated that non-CR after induction was the only independent adverse prognostic factor for OS (HR = 5.665, 95% CI 1.722–18.641, *P* = 0.004). For EFS, three independent factors were identified: platelet count ≥50 × 10^9^/L (protective, HR = 0.156, 95% CI 0.034–0.708, *P* = 0.016), *PTGER4* methylation positivity (adverse, HR = 3.456, 95% CI 1.126–10.610, *P* = 0.030), and non-CR after induction (adverse, HR = 4.480, 95% CI 1.486–13.508, *P* = 0.008). No events occurred in the *CBF*-AML subgroup, leading to unstable hazard ratio (HR) estimates.

#### 3.4.3 Sensitivity analyses for *PTGER4* methylation

To validate the prognostic significance of *PTGER4* methylation and explore potential dose-response relationships, we performed two complementary sensitivity analyses: stratification by methylation percentiles and Cox regression using *PTGER4* as a continuous variable.

Patients were stratified into three groups based on the 25th and 75th percentiles of *PTGER4* CT values: high methylation (CT ≤ 31.09, n = 17), intermediate methylation (31.09 < CT < 45, n = 10), and low methylation (CT ≥ 45, n = 42). Kaplan-Meier analysis revealed a significant dose-dependent effect on EFS, with 3-year EFS rates of 58.8%, 80.0%, and 90.5% across the three groups, respectively (*P* = 0.019, Supplementary Figure S3).

Cox regression analysis further confirmed that *PTGER4* methylation was an independent prognostic factor for EFS when analyzed as a continuous variable (HR = 0.928, 95% CI 0.874–0.986, *P* = 0.015). Each 1-unit increase in *PTGER4* CT value (corresponding to lower methylation) was associated with a 7.2% reduction in event risk. However, *PTGER4* methylation was not an independent prognostic factor for OS in continuous variable analysis (*P* = 0.379). The final multivariate models are presented in Supplementary Table S3.

### 3.5 Subgroup prognostic validation of *PTGER4* methylation

To validate the prognostic value of *PTGER4* methylation in distinct clinical contexts, we first performed stratified survival analyses based on the two independent prognostic factors identified in multivariate Cox regression: platelet count and post-induction remission status (Supplementary Figure S4).

In patients with platelet count <50 × 10^9^/L, *PTGER4* methylation-positive patients had significantly worse OS (*P* = 0.006, Supplementary Figure S4) and EFS (P = 0.008, Supplementary Figure S4). No significant prognostic difference was observed in patients with platelet count ≥50 × 10^9^/L (OS: *P* = 0.160; EFS: *P* = 0.753).

Similarly, in patients who achieved CR after induction therapy, *PTGER4* methylation was associated with significantly inferior OS (*P* = 0.004, Supplementary Figure S4) and EFS (*P* = 0.003, Supplementary Figure S4). In contrast, *PTGER4* methylation status showed no prognostic significance in non-CR (NCR) patients (OS: *P* = 0.753; EFS: *P* = 0.834).

Based on the number of case samples, we conducted further prognostic analyses in another two clinically important subgroups: patients with *KMT2A-r* and subgroups stratified by HSCT receipt, to further characterize the predictive role of *PTGER4* methylation. Within the HSCT subgroup, *PTGER4* methylation was associated with inferior EFS (P = 0.036), while the difference in OS did not reach statistical significance (P = 0.094). Of note, the number of *PTGER4* methylation-positive patients was very limited in non-transplant group and with *KMT2A-r* group (n = 3 for each subgroup). Exploratory analyses suggested worse survival in *PTGER4* methylation-positive cases, but these findings should be interpreted cautiously given the small sample size and were not considered definitive.

### 3.6 Efficacy analysis of hypomethylating agents in *PTGER4* methylation-positive patients

**FIGURE 5:**
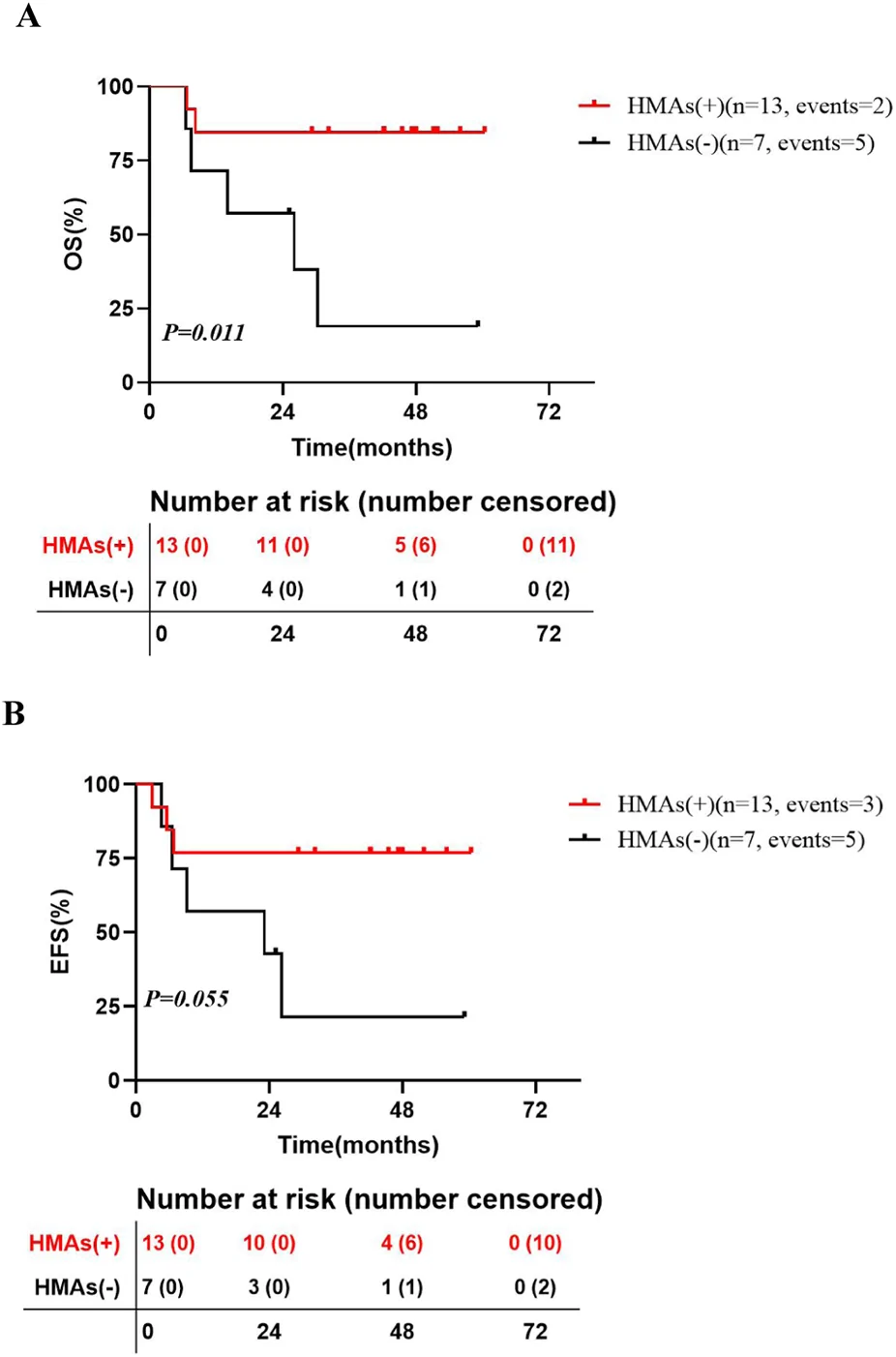
Kaplan-Meier survival curves of OS and EFS in *PTGER4* methylation-positive pediatric AML patients treated with HMAs-based therapy versus conventional chemotherapy. **(A)** OS; **(B)** EFS. AML, acute myeloid leukemia; OS, overall survival; EFS, event-free survival; HMAs, hypomethylating agents.

To evaluate the therapeutic efficacy of HMAs in high-risk patients with *PTGER4* methylation positivity, we compared survival outcomes between patients receiving HMAs ± Ven and those receiving conventional chemotherapy (Figure 5).

Among the 20 *PTGER4* methylation-positive patients, 13 received HMA-based therapy and seven received conventional chemotherapy. Detailed baseline characteristic comparisons between the two groups are presented in Supplementary Table S4. As expected, the HMA group had a numerically higher proportion of high-risk cytogenetics and adverse early treatment response, consistent with the clinical indication for HMA use, though the differences did not reach statistical significance due to the small sample size (all *P* > 0.05). The rate of allo-HSCT was comparable between the two groups.

Kaplan-Meier analysis showed that the 3-year OS rate was significantly higher in the HMA group (84.6% vs. 19.0%, *P* = 0.011, Figure 4). A trend toward improved EFS was also observed in the HMA group (3-year EFS: 76.9% vs. 21.4%, *P* = 0.055).

After adjusting for adverse early treatment response and adverse cytogenetic abnormalities, HMA therapy remained an independent protective factor for OS in *PTGER4* methylation-positive patients (adjusted HR = 0.213, 95% CI 0.058–0.784, *P* = 0.020), confirming the observed survival correlation remained independent after adjustment for baseline high-risk clinical features.

Of note, this survival comparison was exploratory and constrained by the small total number of *PTGER4* methylation-positive patients (n = 20), with merely seven patients allocated to the conventional chemotherapy group. Further subgroup stratification by HMA subtype (azacitidine vs. decitabine, single-agent vs. venetoclax combination) produced excessively small sample sizes per stratum, precluding reliable formal statistical testing. Additionally, only baseline PTGER4 methylation was quantified in our retrospective cohort, without serial epigenetic monitoring during or after HMA treatment. The observed survival association between HMA therapy and improved OS remains correlative and cannot confirm a causal demethylation-driven clinical benefit.

## 4 Discussion

In this study, we systematically evaluated the prognostic value of promoter methylation of three candidate genes (*SHOX2, HOXA9*, and *PTGER4*) in pediatric AML. Our results demonstrated that *PTGER4* promoter methylation was an independent adverse prognostic factor for EFS, with a significant dose-response relationship between methylation levels and survival. Subgroup analyses further confirmed its prognostic utility specifically in patients with platelet count <50 × 10^9^/L and those achieving CR after induction therapy or receiving HSCT. Notably, we observed a survival correlation between HMA-based treatment and longer OS among *PTGER4* methylation-positive patients; this correlative observation requires further mechanistic validation to establish any predictive utility for HMA response.

Consistent with a recent report in adult acute leukemia, we found that *PTGER4* methylation was associated with inferior survival outcomes (Li et al., 2025). However, our study is the first to systematically evaluate the prognostic value of *PTGER4* methylation in pediatric AML and to demonstrate its potential as a predictive biomarker for HMA response. In contrast, neither *SHOX2* nor *HOXA9* methylation showed independent prognostic significance in our cohort, despite their well-documented roles in other malignancies. *SHOX2* methylation is a widely validated diagnostic biomarker in multiple solid tumors (Lu et al., 2022; Krause et al., 2022), while *HOXA9* is a key oncogene driving leukemogenesis through regulation of hematopoietic stem cell self-renewal (Aryal and Lu, 2024; Ye et al., 2023). This may reflect the distinct epigenetic landscapes in pediatric and adult AML (Umeda et al., 2025; Chianese et al., 2023; Zwaan et al., 2026), or differences in patient populations and detection methods.

The dose-response relationship between *PTGER4* methylation levels and EFS further strengthens the biological plausibility of our findings. This indicates that *PTGER4* methylation is not merely a binary marker but a quantitative indicator of disease risk, which may provide more refined risk stratification than simple dichotomization.

The subgroup-specific prognostic value of *PTGER4* methylation has important clinical implications. Our stratified analyses indicated that the prognostic performance of *PTGER4* methylation was population-dependent. In patients with baseline thrombocytopenia, impaired bone marrow reserve may magnify the adverse effects of epigenetic aberrations on disease progression, rendering *PTGER4* methylation capable of identifying an ultra-high-risk subgroup (Park and Yun, 2022). In patients who achieve CR after induction, substantial reduction of overall leukemic burden leaves long-term prognosis predominantly governed by epigenetic features of residual leukemic clones, whereby *PTGER4* methylation retains robust predictive potency for relapse and survival (Schrezenmeier and Huntly, 2025; Xie and Soleimani Samarkhazan, 2025). In contrast, the prognostic effect of *PTGER4* methylation is masked by dominant low-risk genetic features or potent intrinsic chemoresistant factors in low-risk subjects and induction-failure patients, resulting in insignificant survival stratification. Within the HSCT cohort (the main curative therapy for high-genetic-risk pediatric AML), we assessed the prognostic impact of *PTGER4* methylation; *PTGER4* hypermethylation was still associated with inferior EFS after transplantation. This suggests *PTGER4* methylation could further stratify post-transplant relapse risk among patients already classified by genetic risk systems, although consideration of other confounding factors remains warranted.

The tumor-suppressive *PTGER4* gene encodes the EP4 receptor for prostaglandin E2 (PGE2). In normal hematopoietic stem cells, *PGE2-EP4* signaling restricts aberrant self-renewal and promotes myeloid differentiation, limiting the expansion of immature leukemic clones. Promoter hypermethylation of *PTGER4* induces stable transcriptional silencing, ablating this anti-leukemic signaling pathway and enriching chemoresistant leukemic stem cell populations, which ultimately elevates relapse risk and worsens long-term survival in pediatric AML (Markosyan et al., 2024). Hypomethylating agents (azacitidine/decitabine) act by reversing CpG island hypermethylation; theoretically, HMAs can restore *PTGER4* expression and reactivate EP4-mediated tumor suppression, which provides a biological rationale for the observed survival correlation between HMA-based therapy and *PTGER4*-methylated patients in our cohort.

The most clinically relevant finding of our study is the significant survival benefit of HMA-based therapy in *PTGER4* methylation-positive patients. Our results suggest that *PTGER4* methylation may identify a subset of pediatric AML patients who are particularly sensitive to HMAs. Preclinical studies have demonstrated HMAs are capable of reversing promoter hypermethylation to restore *PTGER4* expression, which theoretically could reactivate the *PGE2-EP4* tumor suppressor pathway and sensitize leukemic stem cells to chemotherapy (Li et al., 2026; Mishra et al., 2023). However, we lack serial post-treatment *PTGER4* methylation data in our cohort to verify this mechanistic link *in vivo*. If replicated in larger prospective cohorts with longitudinal epigenetic monitoring, *PTGER4* methylation status may serve as an exploratory correlative marker to inform HMA treatment decisions in pediatric AML. This would enable more personalized treatment decisions, potentially improving survival outcomes while reducing unnecessary toxicity in low-risk patients (Lee et al., 2024; Fleischhacker et al., 2023).

We further contextualize *PTGER4* methylation relative to other commonly studied epigenetic markers (SHOX2, HOXA9, SEPT9, CDKN2A) in terms of prognostic performance, clinical practicability and testing cost. In our cohort, *SHOX2* and *HOXA9* methylation only showed correlations with patient age and did not emerge as independent OS/EFS predictors after multivariate adjustment. Existing literature indicates *SEPT9* and *CDKN2A* perform inconsistently in pediatric AML, without validated pediatric-specific cutoff values. In comparison, *PTGER4* hypermethylation was observed to be an adverse EFS prognostic factor after adjusting for confounders, and appeared to retain risk-stratifying capacity in several high-risk subgroups. From a clinical perspective, *SHOX2* and *HOXA* largely reflect age-associated epigenetic changes and show little potential to inform HMA treatment selection based on current evidence. Our exploratory data suggested *PTGER4* methylation may help identify patients likely to derive survival benefit from HMA consolidation, which could support personalized treatment decision-making. Regarding testing economy, *PTGER4* can be co-detected with *SHOX2* and *HOXA* using one-tube multiplex MS-PCR from routine bone marrow specimens, without extra sampling or additional testing expenses. This platform may be more accessible than costly high-throughput methylation sequencing for routine clinical use. Taken together, compared with previously reported methylation biomarkers that only provide static risk information, *PTGER4* methylation potentially offers dual clinical implications: baseline prognostic stratification and tentative predictive hints for HMA response. It may add complementary value to epigenetic risk evaluation for pediatric AML, though further external validation across larger cohorts is still required.

Our study has several limitations. First, it is a single-center retrospective study with a relatively small sample size, which may limit the generalizability of our findings. Although we have validated the prognostic value of *PTGER4* in some clinical subgroups, due to the limited number of cases, we are unable to conduct a comprehensive and reliable evaluation of the predictive value of *PTGER4* methylation in certain high-risk molecular subgroups. Future multicenter prospective cohort studies are needed to further integrate the *PTGER4* methylation status with standard genetic risk stratification, thereby facilitating the development of a novel epigenetic–genetic combined risk scoring system. Second, the median follow-up time of 43.5 months, while sufficient for evaluating short-term survival, may not capture late relapses beyond 5 years. Third, we did not perform dynamic monitoring of methylation status during treatment or functional experiments to validate the proposed mechanistic hypotheses. Fourth, we prioritized widely recognized consensus cutoffs for baseline platelet count (50 × 10^9^/L) and post-induction MRD (10^−2^) over the cohort-specific optimal thresholds obtained from ROC analysis (52 × 10^9^/L and 0.79%, respectively) to improve comparability with published pediatric AML cohorts, which may slightly weaken risk discrimination performance within our own patient population. Finally, our exploratory analysis of HMA efficacy in *PTGER4* methylation-positive patients is limited by the small sample (n = 20, 7 with conventional chemotherapy), which weakens statistical reliability. No serial post-treatment *PTGER4* methylation data were available to confirm causal epigenetic effects, and stratification by different HMA regimens produced too few cases for robust comparison. As HMA administration was non-randomized based on clinical risk, residual confounding may persist even after multivariate adjustment. These preliminary correlative results need further validation in large prospective cohorts with longitudinal methylation testing.

Additionally, disclosure of methodological details in this study is incomplete in certain aspects. First, the primer sequences, amplicon lengths, and promoter region information for the *SHOX2, HOXA9,* and *PTGER4* gene panel cannot be fully disclosed due to an ongoing patent application (Application No. 202611013302.1; Docket No. BJYJZAX2026023F), which may limit assessment of assay reproducibility by other researchers. Primer sequences are available upon email request to the corresponding author. Second, the assay was designed as a qualitative test yielding dichotomous results based on predefined cut-off values; no standard curve was established. Moreover, commercial plasmid standards for the promoter methylation regions of these three genes are currently unavailable, precluding a linear conversion between CT values and methylation frequencies. The plasmid reference data from our patent application (CT values 37.11–40.62 at 100 copies/μL; 21.86–25.65 at 2000 copies/μL) require further validation in clinical bone marrow samples.

In conclusion, our study demonstrates that *PTGER4* promoter methylation may be a promising independent prognostic biomarker for pediatric AML, with particular utility in patients with baseline thrombocytopenia and those achieving CR after induction or receiving HSCT. Most importantly, *PTGER4* methylation may correlate with response to HMA-based therapy, providing a basis for personalized treatment decisions. These correlative observations require validation in large multicenter prospective cohorts with serial post-treatment methylation detection. At present, *PTGER4* methylation can only be regarded as an exploratory prognostic marker, and additional mechanistic research is required before it can be applied to guide clinical treatment selection.

## Data Availability

The original contributions presented in the study are included in the article/Supplementary Material, further inquiries can be directed to the corresponding author.
