# Supplementary material for: PTGER4 promoter methylation may serve as an independent prognostic biomarker and is associated with response to hypomethylating agents in pediatric acute myeloid leukemia
Source: Front Pharmacol. 2026 Sep 8;17:1922446. doi: 10.3389/fphar.2026.1922446 (PMC13593862; doi:10.3389/fphar.2026.1922446)
Supplement: Supplementary file 1 [file Supplementaryfile1.docx]

**Supplementary Figure 1. Concentration distribution of bone marrow DNA samples for methylation detection (quality control).**

**A**

**B**

**C**

**D**

**Supplementary Figure 2. Distribution of age, white blood cell count, hemoglobin level and platelet count in pediatric AML patients stratified by SHOX2, HOXA9, and PTGER4 methylation status. (A)** WBC; **(B)** Platelet; **(C)**HGB; **(D)** Age.

AML, acute myeloid leukemia; WBC, white blood cell; HGB, hemoglobin; (+), methylation-positive; (−), methylation-negative.

**A**

**B**

**Supplementary Figure 3. Kaplan-Meier curves of OS and EFS in pediatric AML patients stratified by *PTGER4* methylation levels. (A)** OS; **(B)** EFS.

AML, acute myeloid leukemia; OS, overall survival; EFS, event-free survival.

**A**

**B**

**C**

**D**

**E**

**F**

**G**

**H**

**Supplementary Figure 4. Stratified Kaplan-Meier survival curves of OS and EFS in pediatric AML patients according to *PTGER4* methylation status by key clinical subgroups.** **(A)** OS in patients with platelet count ≥50×10⁹/L; **(B)** OS in patients with platelet count <50×10⁹/L; **(C)** EFS in patients with platelet count ≥50×10⁹/L; **(D)** EFS in patients with platelet count <50×10⁹/L; **(E)** OS in patients with post-induction CR; **(F)** OS in patients with post-induction NCR; **(G)** EFS in patients with post-induction CR; **(H)** EFS in patients with post-induction NCR.

AML, acute myeloid leukemia; OS, overall survival; EFS, event-free survival; CR, complete remission; NCR, non-complete remission; (+), methylation-positive; (−), methylation-negative.

**Supplementary Table 1. Comparison of *SHOX2, HOXA9*, and *PTGER4* methylation CT values across clinical and genetic subgroups in pediatric AML**

| Characteristic | No. of Patients  (n) | SHOX2_CT Median (IQR) | P | HOXA9_CT Median (IQR) | P | PTGER4_CT Median (IQR) | P |
| --- | --- | --- | --- | --- | --- | --- | --- |
| With *CBF* |  |  | 0.009 |  | 0.052 |  | 0.004 |
| Absent | 47 | 45.0 (30.94-45.0) |  | 45.00 (31.99-45.00) |  | 45.00 (27.74-45.00) |  |
| Present | 22 | 45.0 (45.0-45.0) |  | 34.06 (26.83-40.79) |  | 45.00 (45.00-45.00) |  |
| With 11q23/  *KMT2A-r* |  |  | 0.709 |  | 0.034 |  | 0.224 |
| Absent | 54 | 45.00 (42.52-45.00) |  | 35.27 (27.03-45.00) |  | 45.00 (29.33-45.00) |  |
| Present | 15 | 45.00 (45.00-45.00) |  | 45.00 (33.48-45.00) |  | 45.00 (36.20-45.00) |  |
| CR after IT |  |  | 0.638 |  | 0.654 |  | 0.686 |
| Yes | 57 | 45.0 (45.0-45.0) |  | 45.0 (27.3-45.0) |  | 45.0 (27.9-45.0) |  |
| No | 12 | 45.0 (40.0-45.0) |  | 36.1 (30.4-45.0) |  | 45.0 (31.5-45.0) |  |
| MRD ≥10^–2^ after IT |  |  | 0.791 |  | 0.338 |  | 0.468 |
| Yes | 21 | 45.0 (38.0-45.0) |  | 34.9 (27.0-45.0) |  | 45.0 (27.8-45.0) |  |
| No | 48 | 45.0 (45.0-45.0) |  | 38.4 (32.1-45.0) |  | 45.0 (32.2-45.0) |  |

AML, acute myeloid leukemia; CT, cycle threshold; IQR, interquartile range; CBF, core binding factor; KMT2A-r, lysine methyltransferase 2A gene rearrangement; CR, complete remission; IT, induction therapy; MRD, minimal residual disease.

**Supplementary Table 2. Spearman correlation between gene methylation CT values and clinical laboratory indicators in pediatric AML**

| Clinical Variable | SHOX2_CT  rₛ (P) | HOXA9_CT  rₛ (P) | PTGER4_CT  rₛ (P) |
| --- | --- | --- | --- |
| Age | -0.264 (0.028) | -0.479 (<0.001) | 0.164 (0.178) |
| WBC | 0.294 (0.014) | 0.301 (0.012) | -0.108 (0.378) |
| Hb | 0.252 (0.037) | 0.103(0.399) | -0.101(0.409) |
| Platelet count | -0.091 (0.457) | 0.108 (0.376) | 0.036 (0.768) |

AML, acute myeloid leukemia; CT, cycle threshold; rₛ, Spearman correlation coefficient; WBC, white blood cell; Hb, hemoglobin.

Note: Negative rₛ values indicate higher methylation levels (lower CT values) with increasing clinical variable values.

**Supplementary Table 3. Multivariate Cox regression analysis of prognostic factors for EFS and OS with *PTGER4* as a continuous variable in pediatric AML**

| Variable | EFS |  | OS |  |
| --- | --- | --- | --- | --- |
|  | HR (95%CI) | P | HR (95%CI) | P |
| Platelet ≥50×10⁹/L | 0.278 (0.076-1.018) | 0.053 | 0.318(0.067-1.508) | 0.207 |
| *PTGER4* CT (continuous) | 0.928 (0.874-0.986) | 0.015 | 0.966(0.907-1.208) | 0.379 |
| Non-CR post-induction | 3.925 (1.278-12.061) | 0.017 | 0.177 (0.054-0.581) | 0.004 |
| MRD ≥ 10^-2^ post-induction | 3.004(0.550-16.395) | 0.278 | 1.878(0.200-17.673) | 0.623 |
| *CBF*-AML | 0(0- ^͚͚^ ) | 0.949 | 0(0- ^͚͚^ ) | 0.960 |

AML, acute myeloid leukemia; EFS, event-free survival; OS, overall survival; HR, hazard ratio; CI, confidence interval; CT, cycle threshold; CR, complete remission; MRD, minimal residual disease; CBF-AML, core binding factor-acute myeloid leukemia.

**Supplementary Table 4: Baseline characteristics of *PTGER4* methylation-positive patients stratified by HMA treatment**

| Characteristic | HMAs group (n=13) | Conventional chemotherapy group (n=7) | P |
| --- | --- | --- | --- |
| Age, years, median (range) | 3 (0.6-15) | 11 (1-15) | 0.152 |
| WBC, ×10⁹/L, median (range) | 9.5 (1.5-165.4) | 15.95 (1.9-228.5) | 0.285 |
| Platelet <50×10⁹/L, n (%) | 5 (38.5) | 3 (42.9) | 1.000 |
| Adverse cytogenetics, n (%) | 4 (30.8) | 2 (28.6) | 1.000 |
| *CBF*-AML, n (%) | 1 (7.7) | 0 (0.0) | 1.000 |
| Non-CR after induction, n (%) | 4 (30.8) | 1 (14.3) | 0.613 |
| MRD ≥10⁻² post-two cycles, n (%) | 5 (38.5) | 2(28.6) | 0.672 |
| HSCT at CR1, n (%) | 10 (76.9) | 5 (71.4) | 1.000 |

HMA, hypomethylating agent; WBC, white blood cell; CBF-AML, core binding factor-acute myeloid leukemia; CR, complete remission; MRD, minimal residual disease; HSCT, hematopoietic stem cell transplantation.
